# Methodological and Quality Flaws in the Use of Artificial Intelligence in Mental Health Research: Systematic Review

**DOI:** 10.2196/42045

**Published:** 2023-02-02

**Authors:** Roberto Tornero-Costa, Antonio Martinez-Millana, Natasha Azzopardi-Muscat, Ledia Lazeri, Vicente Traver, David Novillo-Ortiz

**Affiliations:** 1 Instituto Universitario de Investigación de Aplicaciones de las Tecnologías de la Información y de las Comunicaciones Avanzadas Universitat Politècnica de València Valencia Spain; 2 Division of Country Health Policies and Systems World Health Organization, Regional Office for Europe Copenhagen Denmark

**Keywords:** artificial intelligence, mental health, health research, review methodology, systematic review, research methodology, research quality, trial methodology

## Abstract

**Background:**

Artificial intelligence (AI) is giving rise to a revolution in medicine and health care. Mental health conditions are highly prevalent in many countries, and the COVID-19 pandemic has increased the risk of further erosion of the mental well-being in the population. Therefore, it is relevant to assess the current status of the application of AI toward mental health research to inform about trends, gaps, opportunities, and challenges.

**Objective:**

This study aims to perform a systematic overview of AI applications in mental health in terms of methodologies, data, outcomes, performance, and quality.

**Methods:**

A systematic search in PubMed, Scopus, IEEE Xplore, and Cochrane databases was conducted to collect records of use cases of AI for mental health disorder studies from January 2016 to November 2021. Records were screened for eligibility if they were a practical implementation of AI in clinical trials involving mental health conditions. Records of AI study cases were evaluated and categorized by the International Classification of Diseases 11th Revision (ICD-11). Data related to trial settings, collection methodology, features, outcomes, and model development and evaluation were extracted following the CHARMS (Critical Appraisal and Data Extraction for Systematic Reviews of Prediction Modelling Studies) guideline. Further, evaluation of risk of bias is provided.

**Results:**

A total of 429 nonduplicated records were retrieved from the databases and 129 were included for a full assessment—18 of which were manually added. The distribution of AI applications in mental health was found unbalanced between ICD-11 mental health categories. Predominant categories were Depressive disorders (n=70) and Schizophrenia or other primary psychotic disorders (n=26). Most interventions were based on randomized controlled trials (n=62), followed by prospective cohorts (n=24) among observational studies. AI was typically applied to evaluate quality of treatments (n=44) or stratify patients into subgroups and clusters (n=31). Models usually applied a combination of questionnaires and scales to assess symptom severity using electronic health records (n=49) as well as medical images (n=33). Quality assessment revealed important flaws in the process of AI application and data preprocessing pipelines. One-third of the studies (n=56) did not report any preprocessing or data preparation. One-fifth of the models were developed by comparing several methods (n=35) without assessing their suitability in advance and a small proportion reported external validation (n=21). Only 1 paper reported a second assessment of a previous AI model. Risk of bias and transparent reporting yielded low scores due to a poor reporting of the strategy for adjusting hyperparameters, coefficients, and the explainability of the models. International collaboration was anecdotal (n=17) and data and developed models mostly remained private (n=126).

**Conclusions:**

These significant shortcomings, alongside the lack of information to ensure reproducibility and transparency, are indicative of the challenges that AI in mental health needs to face before contributing to a solid base for knowledge generation and for being a support tool in mental health management.

## Introduction

Mental health represents a vital element of individual and collective well-being, but stressful or adverse living, working, or economic conditions and social inequalities, violence, and conflict can put it at risk. The COVID-19 pandemic has demonstrated how vulnerable mental health can be. Mental health conditions represent one of the leading causes of suffering and disability in the European Region. In 2021, over 150 million people in the WHO (World Health Organization) European Region lived with a mental health condition, and only 1 in 3 people with depression receive the care they need. To address these gaps in mental health services and support, many of which have been exacerbated by the pandemic, WHO/Europe launched a new Pan-European Mental Health Coalition [1]. Mental health is a top priority for the WHO and is a flagship initiative of the European Programme of Work 2020-2025 [2].

Artificial intelligence (AI) has been increasingly used to provide methods and tools for improved diagnosis and treatment of diseases since 2010. AI is defined as the reproducibility of human-like reasoning and pattern extraction to solve problems [3]. AI involves a variety of methods that expand traditional statistical techniques. AI can find patterns that support decision making and hypotheses validation. AI offers a new scope of powerful tools to automate tasks, support clinicians, and deepen understanding of the causes of complex disorders. AI’s presence and potential in health care are rapidly increasing in recent years. AI models need to be fed with the adequate data to be integrated in the clinical workflow and ensuring data quality is crucial [4]. Digitized data in health care are available in a range of formats, including structured data such as electronic health records or medical images, and nonstructured schemas, such as clinical handwritten notes [5].

Because of the possibilities AI offers, policymakers may gain insight into more efficient strategies to promote health and into the current state of mental disorders. However, AI often involves a complex use of statistics, mathematical approaches, and high-dimensional data that could lead to bias, inaccurate interpretation of results, and overoptimism of AI performance if it is not adequately handled [6]. Further, several lacking areas cause concern: transparent reporting in AI models that undermine replicability, potential ethical concerns, validation of generalizability, and positive collaboration in the research community [7,8].

The goals of this review are to map the applications of AI techniques in mental health research, reveal the prominent mental health aspects in this framework, and to assess the methodological quality of the recent scientific literature and evolution of this field in the last 5 years. Systematic reviews and meta-analyses (PRISMA [Preferred Reporting Items for Systematic Reviews and Meta-Analyses] 2020 statement) [9] will be used to design the search strategy and to funnel selection in this systematic overview.

## Methods

### Search Strategy

A systematic literature search was conducted on clinical trials on mental health disorders involving AI techniques using 4 electronic databases: PubMed, Scopus, IEEE Xplore, and Cochrane (Table 1). Search string queries are detailed in Appendix S1 in Multimedia Appendix 1.

### Inclusion and Exclusion Criteria

We specified 3 inclusion criteria for screening. Records were included if they reported a clinical trial (either interventional or observational), were related to mental health disorders, and featured an application of AI. For the final eligibility assessment, exclusion criteria were defined to constrain the review: the reported AI case is not applied for a mental health outcome (ie, applying tools to improve image quality), the record was not published in English, or the report was not published in the last 5 years to review the specific application of these techniques in clinical mental health research. These criteria were designed to evaluate the researching lines in mental health disorders in the last few years, which include the democratization of frameworks and tools for AI application.

**Table 1:** Databases consulted and filters related to our search criteria applied in the search engines.

Database	Filters in search engine
PubMed	Article type: Clinical trial Language: English Publication date: 5 years
Scopus	Document type: Article Language: English
IEEE Xplore	Range years: 2016-2021
Cochrane	Type: Trials Publication date: 2016-2021 Language: English

### Selection Process

**Figure 1:**
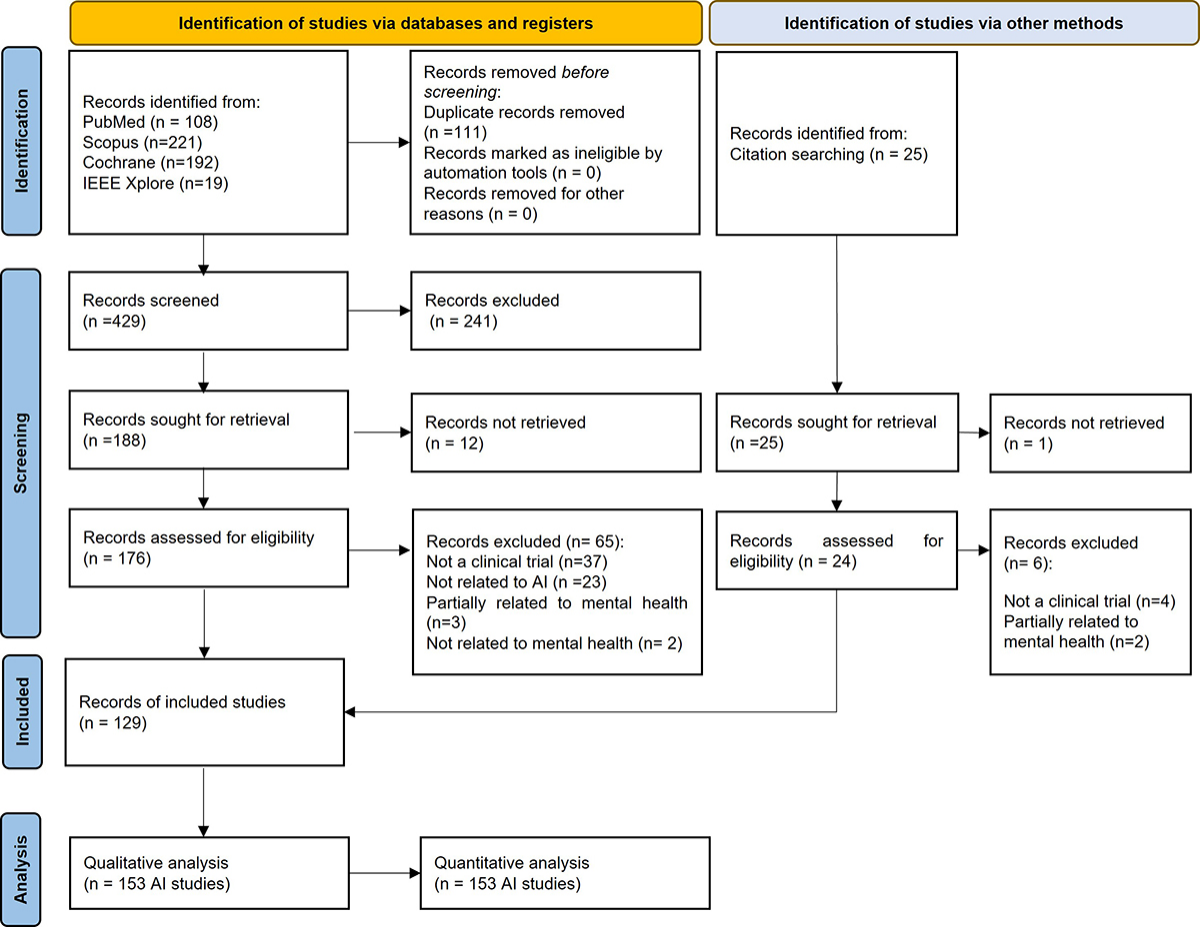
Selection process: PRISMA (Preferred Reporting Items for Systematic Reviews and Meta-Analyses) 2020 flow diagram. AI: artificial intelligence.

Figure 1 shows the flow diagram of the selection process. Records from the scientific literature were identified in the 4 databases defined in Table 1. The resulting data sets were combined in a Microsoft Excel spreadsheet (.xlsx), rearranged by DOI (digital object identifier), and checked for possible erroneous entries. Duplicated records were assessed by comparing DOI names and titles of the publication. A simple code in R 4.2.0 win32 (R Foundation for Statistical Computing) was used to find and tag records whose DOI name and title were already found in the database. The results were manually reviewed to correct minor errors due to misspellings of DOI or the title in the record database. The eligibility criteria for inclusion were then manually evaluated by the title and abstract of each record, and selected records were sought for retrieval. Retrieved records were fully screened and were dismissed if they did not meet the inclusion criteria or met the exclusion criteria. Finally, data and details were extracted for included AI studies.

### Data Extraction

For the included AI applications, 11 categories and 35 data indicators are reported. These indicators were adapted from the CHARMS (Critical Appraisal and Data Extraction for Systematic Reviews of Prediction Modelling Studies) guideline [10]. In addition, publication-related data such as author(s), title, journal, year of publication, and DOI were extracted for the analysis.

Categories were designed to evaluate the goal of the clinical trial and AI, the accessibility and quality of the development process of the data, how the AI model was designed and developed, results, and the reported discussion. Not only are the categories for data extraction designed for detailing the AI models, but also they evaluate the differences and linkages between trial design, data collection, and AI implementation. For further details, see Appendix S2 in Multimedia Appendix 1.

### Quality Assessment

Risk of bias was evaluated by combining the Cochrane tool for randomized controlled trials [11] and PROBAST (Prediction Model Risk of Bias Assessment Tool) guidelines [12]. The Cochrane Handbook for Systematic Reviews of Interventions [13] accounted for the trial design and whether masking and blindness were applied or should have been. The PROBAST guidelines accounted for the suitability of the methodology for collecting the data, candidate predictors, and outcome definition for the AI model as well as how the AI model was applied and analyzed. Both guidelines were considered together to evaluate possible biased relations between trial design and AI applicability. Details of the methodology are provided in Appendix S3 in Multimedia Appendix 1.

## Results

### Articles Identified From the Database Searching

The search identified 540 records, all published in English. Excluding 111 duplicates, 429 articles were screened according to the eligibility criteria based on the title and abstract. The screening process concluded with 241 records excluded for not meeting the inclusion criteria. Of those, 188 records were sought for retrieval, with 12 found impossible to retrieve. Thus, 176 records were assessed for eligibility and 65 were excluded due to not being a clinical trial (n=37), not related to AI (n=23), partially related to mental health (n=3), or not related to mental health (n=2). Furthermore, limitations of search queries were minimized as much as possible by manually adding a selection of AI studies that were not found in the search (n=25). Records from this selection were also screened and sought for retrieval and eventually 18 studies were included. Ultimately, 129 records were included in the analysis. A record could involve 2 or more different cases of AI use for a different outcome, from now on referred to as an AI study. A total of 153 AI studies or AI applications were analyzed. Table 2 summarizes the most important information extracted for this systematic review. Details on the final analysis for each study can be found in Multimedia Appendix 2 (see also [14-142]).

Most used private data (n=142), and a small fraction used public data (n=10) or a mix (n=1). Most studies aimed to develop a new model (n=152); only 1 study aimed to validate a current model. No AI study was intended to update a previously developed AI model. Concerning mental health categories based on the International Classification of Diseases 11th Revision (ICD-11), nearly one-half of the studies (77/153, 50.3%) related to mood disorders, which combines the Depressive disorders (n=70) and Bipolar or related disorders (n=4) categories; 3 other studies used data from patients within both categories, labeled as “mood episodes.” The second most common category was Schizophrenia or other primary psychotic disorders (n=26), and the third was Disorders specifically associated with stress (n=12). Some studies included participants with different mental disorders (n=10).

Other categories were Anxiety or fear-related disorders (n=7); Secondary mental or behavioral syndromes associated with disorders or diseases classified elsewhere (n=5); Disorders due to substance use (n=5); Neurocognitive and dementia disorders (n=4); Neurodevelopmental disorders (n=1); Obsessive-compulsive or related disorders (n=1); Feeding or eating disorders (n=1); Bodily distress disorder (n=1); Personality disorders (n=1); and Mental or behavioral disorders associated with pregnancy, childbirth, or the puerperium, without psychotic symptoms (n=1).

**Figure 2:**
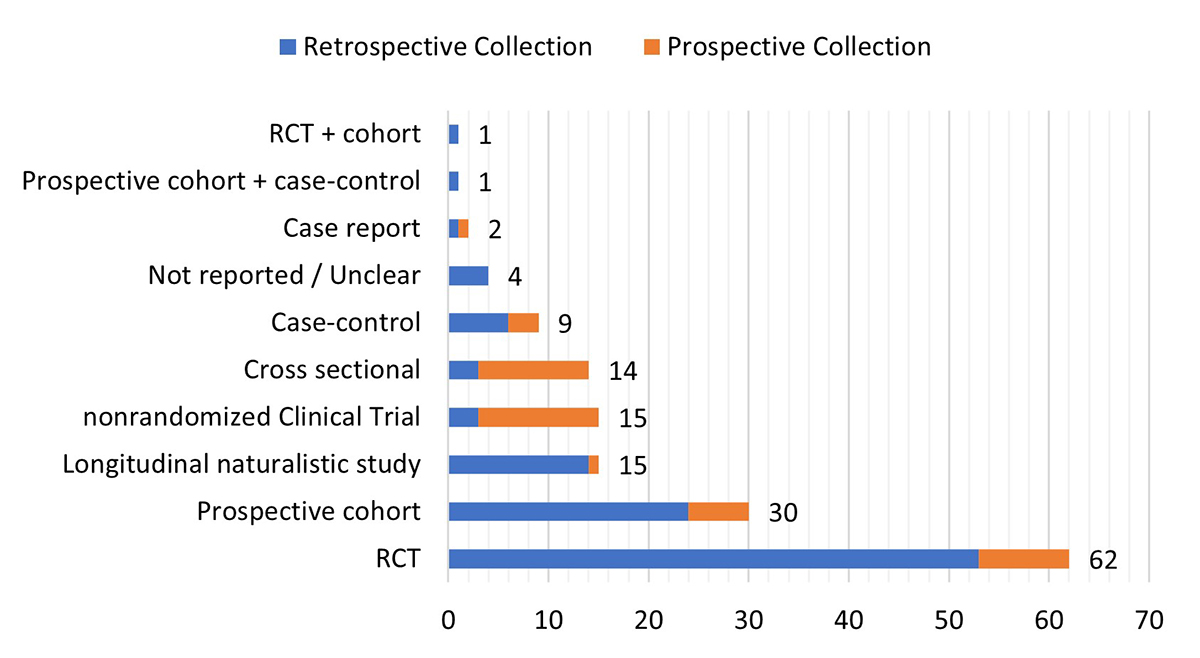
Count of trial designs where data were retrieved. Orange specifies only studies with their own designed trial. RCT: randomized clinical trial.

Only 28.1% (43/153) of studies used original data collected within the study, while 71.9% (110/153) of studies retrieved data from databases or were a secondary analysis of clinical trials not designed for that purpose. The most common type of trial design was randomized clinical trial (n=62), followed by prospective cohort study designs (n=30) and nonrandomized clinical trial designs (n=15). Further, we found longitudinal naturalistic studies (n=15), cross-sectional designs (n=14), case-control designs (n=9), and case reports (n=2). Two reports of AI cases used a mix of trial designs and 4 did not report this or the references were unclear. Figure 2 shows the distribution of study design based on the prospective or retrospective collection of data.

Not all studies reported enough details to evaluate recruitment of participants (n=17). Almost one-half of the studies collected data from different locations (n=75), whereas the rest only reported 1 location (n=61). Of the multisite studies, only one-third used international collection (17/153, 11.1%). Only 13 of the 43 (30%) prospective collection studies followed a multisite collection method (n=13), and only 1 study was international.

**Table 2:** Key summary of artificial intelligence studies (N=153) analyzed (n=129 articles).

Mental health disorder (ICD-11^a^) section number: category	Artificial intelligence model family	Data type
6A0: Neurodevelopmental disorders	Regression [14]	Mixed^b^ [14]
6A2: Schizophrenia or other primary psychotic disorders	Competing models^c^ [15-17] Ensembled models [18,19] Regression [20-23] Statistical clustering [24] SVM^d^ [25-28] Trees [29-31] Regression and statistical clustering [32] Regression and hierarchical clustering [33]	Genomic data [29] Medical image [15-19,21-23,27,28,33] Mixed [20,26,30,32] Questionnaires and scales [24,25,31]
6A6: Bipolar or related disorders	Bayesian [34] Manifold [35] Regression [36] SVM [37]	Medical image [35,37] Mixed [36] Questionnaires and scales [34]
6A7: Depressive disorders	Bayesian [38-40] Competing models [41-53] Deep learning [54] Ensembled models [55-57] Hierarchical clustering [58,59] Markov model [60] Mixture model [61] Mixture model, regression, and trees [62] Regression [63-74] Relevance vector machine [75] Statistical learning [76,77] SVM [78-86] Trees [87-93] Trees and hierarchical clustering [94] Trees and statistical learning [95]	Audio recording [64] Biomarkers [51,54,95] Biosignal [41] I^e^ [80,81,87,93] Genomic data [44,70,74] Medical image [38,40,48,67-69,75,82,85] Mixed [42,43,45,46,49,50,52,53,55-57,59, 61,63,65,66,71-73,77,79,83,86,88-92,94] Questionnaires and scales [47,58,60,62,76] Text [39] Video image [78,84]
6A6, 6A7: Mood episodes	Competing models [96] SVM [97] Regression [98]	Medical image [97,98] Questionnaires and scales [96]
6B0: Anxiety or fear-related disorders	Competing models [99-101] Regression [102] SVM [103] Trees [104]	Biomarkers [101] Biosignal [100] Mixed [99,103,104] Text [102]
6B2: Obsessive-compulsive or related disorders	Competing models [105]	I [105]
6B4: Disorders specifically associated with stress	Competing models [106] Ensembled models [107,108] Hierarchical clustering [109] Mixture model and regression [110] SVM [111-115] Trees [116]	Audio recording [116] Biosignal [115] Medical image [112-114] Mixed [106-108,110,111] Questionnaires and scales [109]
6B8: Feeding or eating disorders	Competing models [117]	Mixed [117]
6C2: Bodily distress disorder	Regression [118]	Mixed [118]
6C4: Disorders due to substance use	Competing models [119] Regression [120] Trees [121,122]	Medical image [120] Mixed [119,121,122]
6D1: Personality disorders	Trees [123]	Mixed [123]
6D7, 6D8: Neurocognitive disorders and dementia	Competing models [124] Ensembled models [125,126] Trees [127]	Mixed [124-127]
6E2: Mental or behavioral disorders associated with pregnancy, childbirth, or the puerperium, without psychotic symptoms	Competing models [128]	Mixed [128]
6E6: Secondary mental or behavioral syndromes associated with disorders or diseases classified elsewhere	Bayesian [129] Competing models [130] Regression [131] Trees [132,133]	Mixed [130-133] Questionnaires and scales [129]
Combination of some ICD-11 categories in mental health	Bayesian [134] Ensembled models [135-137] Regression [138,139] Regression and support vector machines [140] Trees [141]	Biosignal [140] Mixed [135-137,139] Questionnaires and scales [134,138,141]
Unspecified^f^	Competing models [142]	Mixed [142]

^a^ICD-11: International Classification of Diseases 11th Revision.

^b^Mixed: combination of type of data and predictors.

^c^Competing models: the study was designed for evaluate several types of artificial intelligence model families without assessing a priori adequacy.

^d^SVM: support vector machine.

^e^I: electronic health records.

^f^Unspecified: The outcome of the study is “mental health problems,” therefore, it could not be classified in any specific category.

### AI Applications

Studies were categorized according to the intended use of the AI models in the research. The most common category was studies for evaluating treatments, Treatment quality (n=44), followed by Subgroups/patterns identification (n=31), Predictor identification (n=28), Prognosis (n=23), Diagnose (n=20), and Forecasting symptoms (n=7).

Most Treatment quality applications used retrospective data from previous randomized clinical trials (n=28) and in these studies the clinical arms were treated as different cohorts to compare AI outcomes and performance (n=28). The same results can be found in Predictor identification, where close to one-half of the studies collected data from previous randomized clinical trials to compare different clinical arms (n=13). In the Subgroups/patterns identification category, studies collected data from a balanced mix of study designs, while in Prognosis the most common method was prospective cohort studies. In the Diagnose and Forecasting symptoms studies, none of the categories stood out in particular. More detailed results are presented in Appendix S4 in Multimedia Appendix 1.

**Figure 3:**
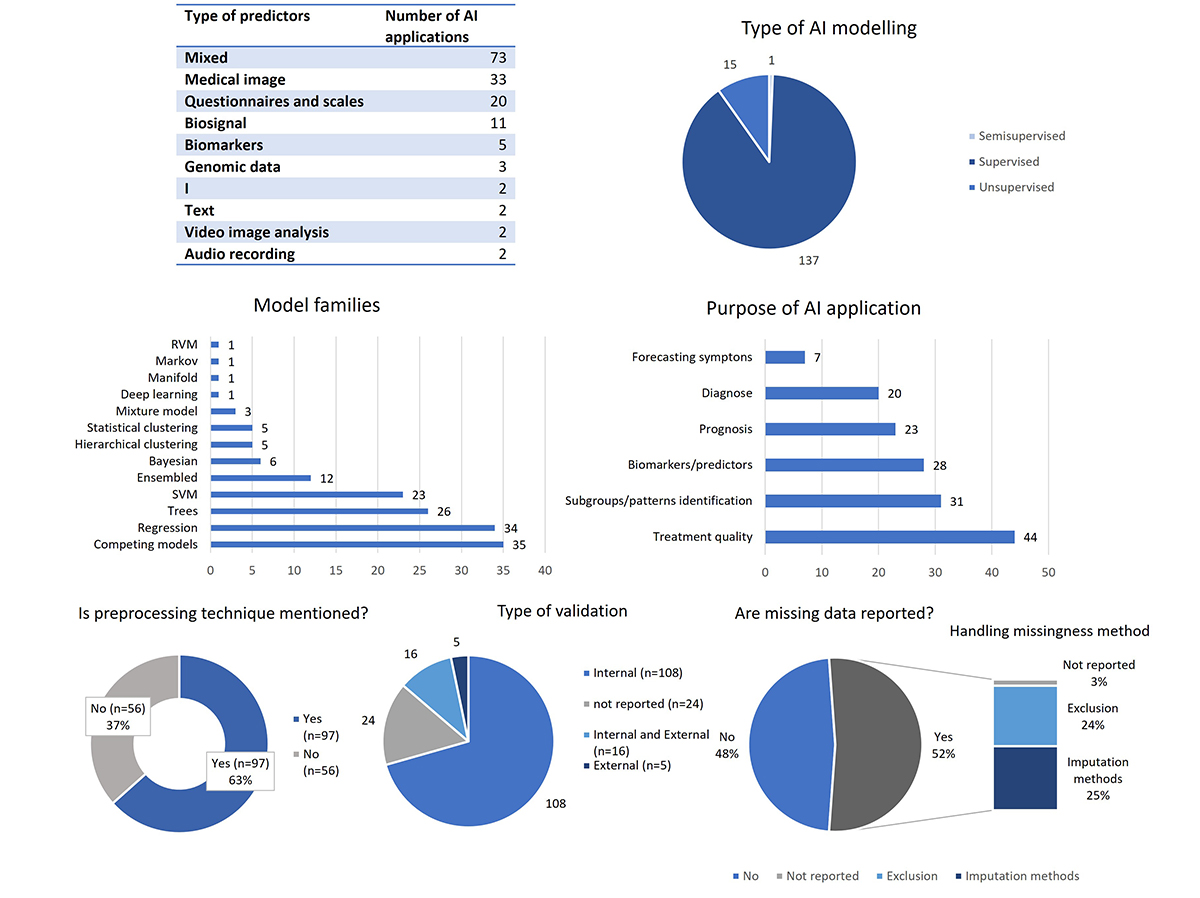
Dashboard and descriptive analytics on AI developing and preprocessing pipeline. AI: artificial intelligence; EHR: electronic health record; RVM: relevance vector machine; SVM: support vector machine.

Figure 3 presents a dashboard that summarizes the AI model results regarding candidate predictors, preprocessing pipeline, AI techniques, and validation. For candidate predictors, many studies used a combination of data (n=73). The most individually common category was Medical image (n=33), which relates to medical imaging analysis (ie, region of interest or voxel-based morphometry), and the second was Questionnaires and scales (n=20), defined as any self-reported or interview-reported scale for symptom severity, conditions, or actual mood. The third was Biosignal collection (n=11), such as electroencephalography or electrocardiography and related analyses. Other data categories were Biomarkers (n=5), Genomic data (n=3), Electronic health records or I (n=2), Text (n=2), Video image analysis (n=2), and Audio recording (n=2). I refers to historical, demographic, and clinical information collected in hospitals and specialty care sites. Text refers to any data that are used for natural language processing analysis, such as written text or speech. Audio recording was introduced as the analysis of audio and voice features unrelated to language processing. The Mixed category (n=73 studies) combined data from I and different questionnaires and symptom scales (49/73, 67%); the remaining studies included other categories such as Biomarkers (n=7), Medical image (n=4), Genomic data (n=3), Biosignal (n=3), and Text (n=2). Medical image was also combined with Genomic data (n=1) and Biomarkers (n=1). Besides, Biomarkers were combined with Questionnaires and scales (n=2) and with Biosignals (n=1).

When evaluating data quality, methods to assess data suitability, and preprocessing pipelines, only 12/153 studies (7.8%) considered the statistical power of the sample size; 37.3% (57/153) of studies used a sample size of 150 or less to train the AI models. Only 13.7% (21/153) reported external validation (n=5) or reported both internal and external validation (n=16). The rest of the AI studies used only internal validation (n=108) or did not report the validation method (n=24). Only 38.6% (59/153) of studies reported a method to assess significance of their performance results, while the majority did not detail any (n=94).

AI studies used supervised learning, semisupervised learning, and unsupervised learning methods. No reinforcement learning algorithms were found. Regarding AI algorithms, the most popular family of techniques was regression (n=34), followed by trees (n=26) and support vector machine (n=23), which constitutes most AI studies. Other algorithms were Bayesian (n=6), statistical clustering (n=5), hierarchical clustering (n=5), mixture model (n=3), deep learning (n=1), manifold (n=1), Markov model (n=1), and relevance vector machine (n=1). In some cases, an Ensembled model was designed with the inclusion of different types of AI algorithms (n=12). Another category, Competing models (n=35), refers to the AI studies that did not predefine a specific AI technique or algorithm based on features of the data and instead applied different techniques with the intention to retain the model algorithm with the best performance to their outcome definition. These 35 studies used 144 AI techniques in total.

Regarding preprocessing methods, only 63.4% (97/153) of studies reported whether they applied any preprocessing technique to data or that preprocessing was not needed, while the rest did not report any (n=56). Regarding data gaps, only 52.3% (80/153) of studies reported or mentioned if there were some missing data in samples or not, while 47.7% (73/153) did not. Of the studies reporting missing data, 2.6% (4/153) did not report any method to handle missing data bias, whereas 24.2% (37/153) opted for excluding the samples and 25.5% (39/153) chose to impute the missing values from the data distribution by different imputation methods. Of these, only 2 studies detailed the type of missingness. The proportion of reporting missing data was similar for both retrospective and prospective data collection.

### Risk of Bias and Transparency

**Figure 4:**
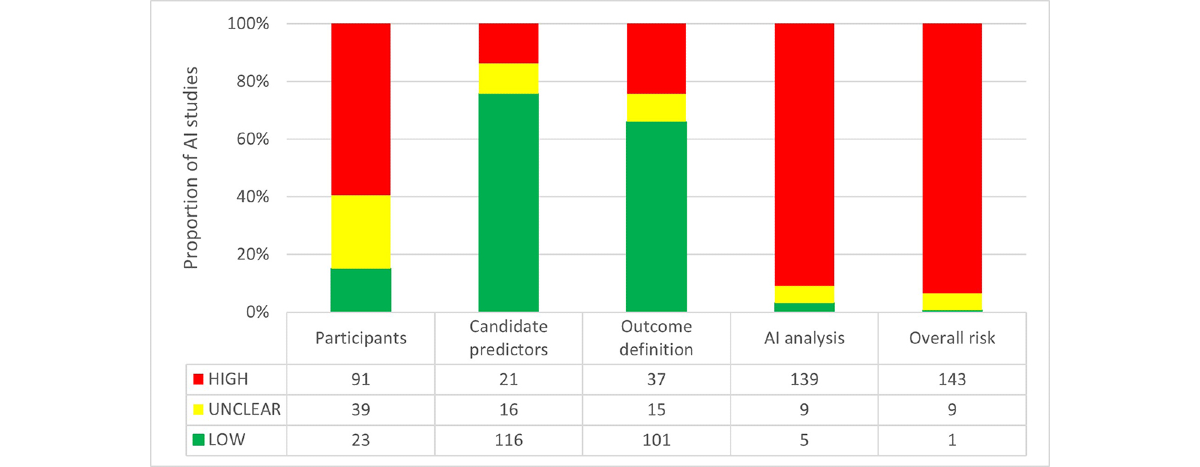
Analysis of the risk of bias following PROBAST (Prediction Model Risk of Bias Assessment Tool) categories as defined in Multimedia Appendix 1. AI: artificial intelligence.

The risk of introducing bias is defined in Appendix S3 in Multimedia Appendix 1. Only 23 studies were found unlikely to introduce bias due to the trial design and evaluation of participants, whereas in the majority of studies the risk was high or unclear. In most cases, the risk of bias due to participants and the trial features was introduced by bias in the distribution of participants—that is, inclusion and exclusion criteria, loss of follow-up, and participants withdrawal—or the sample was not enough to be considered a good example of the target population. The definition and collection of the candidate predictors were mostly a low risk of introducing bias (n=16), with a few studies possibly introducing bias (n=21) or having an unclear risk of bias (n=16). Results for the outcome definition in the AI model are similar, with most studies evaluated as having a low risk of bias (n=101). Some studies were categorized as unclear (n=15) or high risk (n=37) due to unclear definitions of outcomes or combining data set of different populations whose outcomes were evaluated with different methods. The most important risk of bias was found when applying AI algorithms and their evaluation. Only a few studies were evaluated as unlikely to introduce some bias (n=5) and the vast majority of AI analysis introduced a high risk of bias (n=139), while 9 could not be assessed properly (n=9). The main issues for bias in the AI analysis were not appropriately preprocessing and arranging the data for the specifications of the applied AI model, a bad handling of missingness, or an insufficient validation of the performance to account for overfitting and optimism (Figure 4). Appendix S5 in Multimedia Appendix 1 shows a stratified analysis of the risk of bias based on disorders, study designs, and outcome.

**Figure 5:**
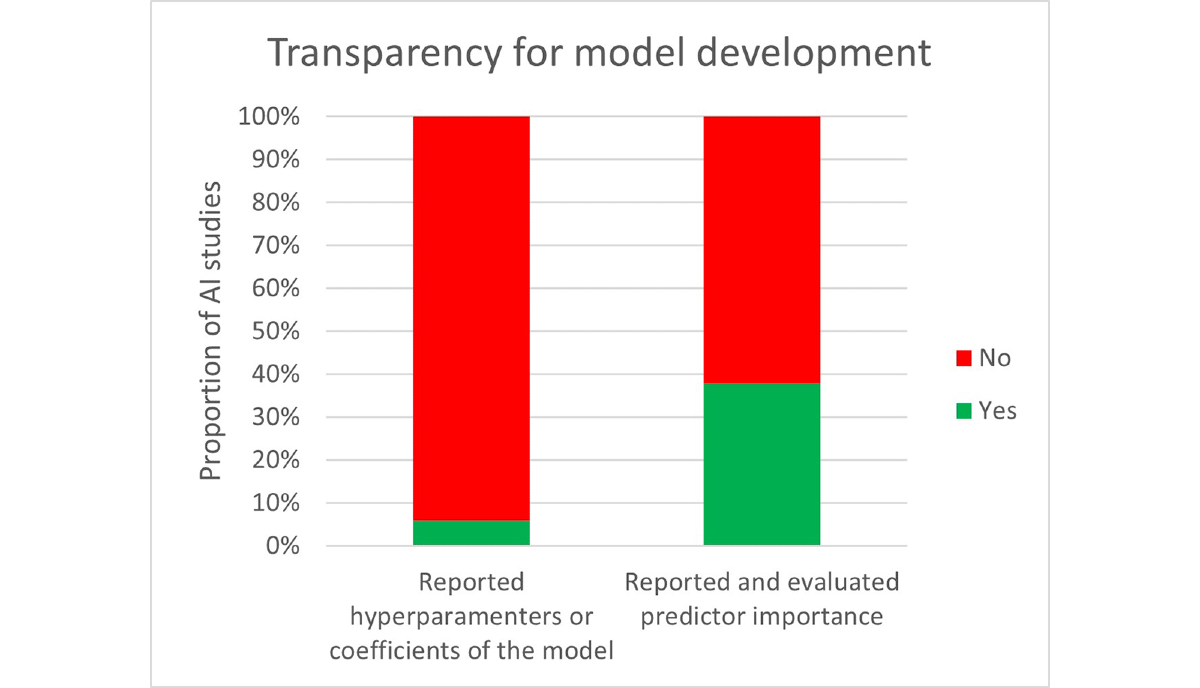
Reporting of candidate predictor importance as well as hyperparameters for model tuning and coefficients of models. AI: artificial intelligence.

Overall, only 1 AI study could be assessed as a low risk of being biased. The most contributing categories to the overall risk were Participants and AI analysis. Most studies were likely unbiased about the definition and collection of predictors and the outcome but they failed to apply these data later in the model—bad data engineering or bad validation of the models—or the trial design had some flaws from the beginning. It is worth mentioning that only 9 of the 153 models reported any hyperparameter tuning or coefficients of the models and most of them reported basic trees models coefficients and decision rules. Only 58 studies mentioned or reported predictor importance and less than one-half reported the ranking and evaluated the methodology to test it (Figure 5).

## Discussion

### Principal Findings

This overview summarizes the development and status of AI applications in mental health care. The review focused on the period from 2016 to 2021 to understand the latest advancements of these applications in mental health research, including aspects related to the methodological quality, the risk of bias, and the transparency. Results may be limited by the keywords applied in the search queries. This analysis is only representative for the records retrieved in this search. However, the samples analyzed may be sufficient to judge the quality and current status of this field. Significant methodological flaws were found involving the statistical processes of AI applications and data preprocessing. Many studies overlook evaluating or reporting on data quality and how it fits the model. Some studies applied several AI techniques, here aggregated as “compelling models” studies, to select the most efficient technique without assessing their suitability for the problem they face, which may lead to overoptimism. Further, missing data management and the recruitment of participants are rarely reported, making it difficult to account for the risk of model overfitting. Preprocessing pipelines are not sufficiently reported, which hampers the reproducibility of the study or the adaptation of the AI techniques to the specific type of study. The use of reporting guidelines, such as the CONSORT-AI (AI version of the Consolidated Standards of Reporting Trials) for clinical trial reports involving AI [143], the SPIRIT-AI (AI version of the Standard Protocol Items: Recommendations for Interventional Trials) for clinical trial protocols involving AI [144], or the MI-CLAIM (Minimum Information About Clinical Artificial Intelligence Modeling) checklist on minimum information for clinical AI modeling [145], would be very useful to ensure that the basic information about the study design and implementation is reported in these types of studies.

Some predictive models are being updated for AI reporting, such as TRIPOD-AI (AI version of the Transparent Reporting of a Multivariable Prediction Model of Individual Prognosis or Diagnosis) and PROBAST-AI (ie, the AI version of the PROBAST) [146]. However, these guidelines are rarely followed in the reviewed records. They lack transparent reporting on AI model features such as coefficients, hyperparameters, or predictor selection. Encouraging transparent reporting should be prioritized, as it would benefit second external validations and provide better accountability for reported models.

The incorporation of AI in mental health research is unbalanced between ICD-11 mental health disorders. Most research focusses on depressive disorders, where it combines severity questionnaires and scales with electronic health record and psychotic disorders using medical image data. External validation is very uncommon. Conducting suitable trial designs for the intended AI outcomes is understandably difficult in terms of money, time, and resources. Thus, it is common to apply data collected retrospectively. However, the original trial designs do not fit the specifications for AI development and most studies do not assess the appropriateness of these data. Notably, many authors may not understand the need to ensure an optimal preprocessing pipeline. In these cases, authors are aware of the poor performance of the models, but the proposed approach for improvement is suggested directly from a trial perspective rather than from assessing possible statistical bias or mistakes in model development, which could save cost and time over designing new studies.

### Challenges

AI studies were analyzed to identify challenges and opportunities involving the use of AI in mental health. Typically, AI studies reported insufficient samples to ensure model generalizability [68,84,103]. Several authors reported bias because of the difficulty in adapting typical trial designs to an AI context. For example, some authors detail the constraining boundaries for selecting participants in randomized clinical trials as a limiting factor, which reduces the sample size and could overlook confounders [68,90]. Most randomized clinical trials noted possible variance between the collected data and the real-world data. However, observational studies can also introduce bias in AI models if the imbalance between cohorts is not adequately addressed [84,128]. In these studies, the variety in features such as prescribed medication could introduce confounders and bias that are difficult to manage [94]. Furthermore, in long-term studies, lack of follow-up or other conditions leading to a decrease of patients is an important limitation, mostly for prognosis studies or predictive evolution of condition severity [30,58]. These issues are worse for retrospective collection of data, where trial designs tend to diverge from the problematics of AI. Besides, some authors are aware of bias due to gaps, but most did not properly evaluate this risk.

A noticeable lack of internationalization was detected. Many studies focused only on local data, which contributes to small sample sizes and poor generalizability [115,127]. Encouraging partnerships and collaborations across countries and centers should be a priority, as it could facilitate external validation [71]. Some authors mention difficulties reconciling clinical practices with AI study requirements, usually due to ethics problems related to clinical practice in patients that can overlook confounders, that is, making it difficult to apply placebo controls in some interventions [82,115].

Another challenge is the explainability of complex AI models, which could make researchers reluctant to adopt techniques that map high-order interactions or “black-box” algorithms [81,122]. Researchers prefer simpler algorithms. The few studies that reported model coefficients and some explanation used decision trees. Another challenge is that contradictory findings could occur among studies [85].

Finally, some authors are aware of the opportunities that everyday devices and platforms such as phones and social networks offer but find it difficult to take advantage of these tools due to lack of standardization, which reduces the target population for defining a study [92].

### Opportunities

Some studies introduced devices and platforms to improve the monitoring of patients. The application of everyday digital tools could reduce necessary resources and therefore facilitate data collection [99,127]. Promoting the use of frequently used devices combined with the application of AI models seems like a future trend that could improve the treatment of many conditions where the chance of treatment response decreases over time [126]. Further, it opens possibilities of internet-based treatments that could be conducted in real time with digital technology, easing the load on hospitals [99].

Data sharing and public databases should be encouraged to develop and implement more trustworthy AI models. AI models from clinical stage to clinical practice could be difficult but powerful tools to gain insights into predictor collection, human-based decisions, and AI biases while these techniques are being implemented in clinical world. Many studies report the high potential of AI in mental health for clinical support, computer-aided systems, and possibly preliminary screening [94,127].

Currently, many guidelines and initiatives exist to which researchers could adhere to in order to increase transparency and better use AI models. Currently, the EQUATOR (Enhancing the Quality and Transparency of Health Research) network initiative reports useful guidelines that could foster collaboration and implementation [147].

### Conclusion

AI algorithms are increasingly being incorporated into mental health research; however, it is still uneven between ICD-11 categories. Collaboration is merely anecdotal, and data and developed models mostly remain private. Significant methodological flaws exist involving the statistical process of AI applications and data preprocessing pipelines. Only 1 study was found reporting second validation, and 13.7% (21/153) reported external validation. The evaluation of the risk of bias and transparent reporting was discouraging. Model hyperparameters or trained coefficients are rarely reported, nor are insights about the explainability of the AI models. The lack of transparency and methodological flaws are concerning, as they delay the safe, practical implementation of AI. Furthermore, data engineering for AI models seems to be overlooked or misunderstood, and data are often not adequately managed. These significant shortcomings may indicate overly accelerated promotion of new AI models without pausing to assess their real-world viability.

## Appendix

Multimedia Appendix 1 Search string queries; description of categories and indicators; evaluation of risk of bias; data collection for AI applications; and risk of bias stratified by disorders, study designs, and outcome. AI: artificial intelligence.

Multimedia Appendix 2 Analysis results.

## Abbreviations

AI: artificial intelligence
CONSORT: Consolidated Standards of Reporting Trials
CHARMS: Critical Appraisal and Data Extraction for Systematic Reviews of Prediction Modelling Studies
DOI: digital object identifier
EQUATOR: Enhancing the Quality and Transparency of Health Research
ICD-11: International Classification of Diseases 11th Revision
IEEE: Institute of Electrical and Electronics Engineers
MI-CLAIM: Minimum Information About Clinical Artificial Intelligence Modeling
PRISMA: Preferred Reporting Items for Systematic Reviews and Meta-Analyses
PROBAST: Prediction Model Risk of Bias Assessment Tool
SPIRIT: Standard Protocol Items: Recommendations for Interventional Trials
SVM: support vector machine
TRIPOD: Transparent Reporting of a Multivariable Prediction Model of Individual Prognosis or Diagnosis
