# Supplementary material for: Methodological and Quality Flaws in the Use of Artificial Intelligence in Mental Health Research: Systematic Review
Source: JMIR Ment Health. 2023 Feb 2;10:e42045. doi: 10.2196/42045 (PMC9936371; doi:10.2196/42045)
Supplement: Multimedia Appendix 1 [file mental_v10i1e42045_app1.docx]

# Appendix

## S1. Search string queries

PUBMED

(("mental health"[Title/Abstract] OR "Depression"[Title/Abstract] OR "Anxiety"[Title/Abstract] OR {Self-Injury}[Title/Abstract] OR "Mental Illness"[Title/Abstract] OR Psychosis[Title/Abstract] OR psychotic[Title/Abstract] or Trauma[Title/Abstract] or "Stress disorder"[Title/Abstract] or Bipolar[Title/Abstract] or "Suicide Prevention"[Title/Abstract] or Dysphoria[Title/Abstract] or "Eating Disorder") AND ("artificial intelligence"[Title/Abstract] OR "machine learning"[Title/Abstract] OR "deep learning"[Title/Abstract] OR "reinforce learning"[Title/Abstract] OR "unsupervised learning"[Title/Abstract] OR "Clustering"[Title/Abstract] OR "Computer Vision"[Title/Abstract] or "Clinical Decision Support"[Title/Abstract])) AND ((y_5[Filter]) AND (humans[Filter]) AND (english[Filter]))

Filters:

- Article type: Clinical trial
- Language: English
- Publication date: 2016 to 2021

SCOPUS

( TITLE-ABS-KEY ( "mental health" ) OR TITLE-ABS-KEY ( depression ) OR TITLE-ABS-KEY ( anxiety ) OR TITLE-ABS-KEY ( "Self-Injury" ) OR TITLE-ABS-KEY ( {Mental Illness} ) OR TITLE-ABS-KEY ( psychosis ) OR TITLE-ABS-KEY ( psychotic ) OR TITLE-ABS-KEY ( trauma ) OR TITLE-ABS-KEY ( "Stress disorder" ) OR TITLE-ABS-KEY ( bipolar ) OR TITLE-ABS-KEY ( "Suicide Prevention" ) OR TITLE-ABS-KEY ( dysphoria ) OR TITLE-ABS-KEY ( "Eating disorder" ) ) AND ( TITLE-ABS-KEY ( "Artificial Intelligence" ) OR TITLE-ABS-KEY ( "Machine Learning" ) OR TITLE-ABS-KEY ( "Deep Learning" ) OR TITLE-ABS-KEY ( "reinforce learning" ) OR TITLE-ABS-KEY ("unsupervised learning" ) OR TITLE-ABS-KEY ( "computer vision" ) OR TITLE-ABS-KEY ( "Clinical Decision Support" ) ) AND ( TITLE-ABS-KEY ( "Clinical Trial" ) OR TITLE-ABS-KEY ( "Random Clinical Trial" ) OR TITLE-ABS-KEY ( {RCT} ) ) AND ( LIMIT-TO ( PUBYEAR , 2021 ) OR LIMIT-TO ( PUBYEAR , 2020 ) OR LIMIT-TO ( PUBYEAR , 2019 ) OR LIMIT-TO ( PUBYEAR , 2018 ) OR LIMIT-TO ( PUBYEAR , 2017 ) OR LIMIT-TO ( PUBYEAR , 2016 ) ) AND ( LIMIT-TO ( LANGUAGE , "English" ) ) AND ( LIMIT-TO ( EXACTKEYWORD , "Human" ) )

Filters

- Document type: Article
- Language: English

IEEE XPLORE

(("Abstract":"mental health" OR "Abstract":"Depression" OR "Abstract":"Anxiety" OR "Abstract":"Self-Injury" OR "Abstract":"Mental Illness" OR "Abstract":"Psychosis" OR "Abstract":"psychotic" OR "Abstract":"Trauma" OR "Abstract":"Stress disorder" OR "Abstract": "Bipolar" OR "Abstract":"Suicide Prevention" OR "Abstract":"Dysphoria" OR "Abstract":"Eating Disorder") AND ("Abstract":"artificial intelligence" OR "Abstract":"machine learning" OR "Abstract":"deep learning" OR "Abstract":"reinforce learning" OR "Abstract":"unsupervised learning" OR "Abstract":"Clustering" OR "Abstract":"Computer Vision" OR "Abstract":"Clinical Decision Support") AND ("Full Text & Metadata":"Clinical Trial" OR "Full Text & Metadata": "Randomized Controlled Trial"))

Filters:

- Range years: from 2016 to 2021

COCHRANE

"mental health" OR "Depression" OR "Anxiety" OR "Self-Injury" OR "Mental Illness" OR "Psychosis" OR "psychotic" OR "Trauma" OR "Stress disorder" OR "Bipolar" OR "Suicide Prevention" OR "Dysphoria" OR "Eating Disorder" in Title Abstract Keyword AND "artificial intelligence" OR "machine learning" OR "deep learning" OR "reinforce learning" OR "unsupervised learning" OR "Computer Vision" OR "Clinical Decision Support" in Title Abstract Keyword - with Publication Year from 2016 to 2021, *in Trials (Word variations have been searched)*

Filters:

- Type: Trials
- Publication date: from 2016 to 2021
- Language: English

## S2. Description of categories and indicators

| Category | | |
| --- | --- | --- |
| Predictor | Description | Type |
| Objective | | |
| ICD-11 3^rd^ level | ICD-11 disorder category at 3º level (subgroups of 6º level [6Lx]). | Categorical |
| ICD Mental Disorders Groups | Definition of subgroups in Mental Health disorders | Categorical |
| AI treatment | Goal of using AI. To develop a new AI model for specific outcome, to validate an existing model, to update or re-develop (applying an already developed model, fine-tuning…) | Categorical |
| Data | | |
| Accessibility | Either Public (free access) or Private (not available or need permissions). | Categorical |
| Data treatment | How the cohorts/trial arms/groups of participants in the trial were treated in the AI model. For example, a second study could subset a specific cohort while ignoring the rest. This can lead to possible bias when the study design was not suited for that treatment. | Categorical |
| Study design | Type of trial from which the data was generated. RTC, prospective cohort, | Categorical |
| Origin | Define if data was collected prospectively reporting a trial designed specially for the AI study or if the AI analysis used data retrieved from databases or were second analysis studies. | Categorical |
| Participants | | |
| Participant recruitment method | How participants were recruited. | Description |
| Multisite | Y/N. Whether participants were recruited from multiple locations in the same country or abroad. | Logical |
| International | Y/N. Whether participants were recruited from different countries based on a international research effort. | Logical |
| Outcome quality | | |
| Purpose of AI application | Goal the AI model was applicated for. Six categories were defined a priori:   - Predictor identification. - Subgroups or pattern identification. - Forecasting symptoms. - Treatment quality. - Prognosis. - Diagnose. | Categorical |
| Was the outcome defined (described) and used consistently in all patients (outcome and measure)? | Y/N. If outcome is clearly defined as an objective measure and if this measure is applied for all patients. | Logical |
| Single or combined outcome? | If outcome is specific to one disorder or definition or it is a combination (i.e. vascular diseases) | Categorical |
| Blinded outcome? (Y/N) | If candidate predictors in AI model were not used to assess the actual outcome by researchers/physicians. | Logical |
| Candidate predictors | | |
| Type of predictors | Audio recordings, Biomarkers, Biosignal, EHR, Genomic data, Medical image, Mixed, NLP input, Questionnaries and scales, Video image | Categorical |
| Is data preprocessing metioned? | Y/N. Whether data preprocessing is mentioned in the publication. It is possible a publication mentioned data was preprocessed for the AI model but never explain the details of the preprocessing. | Logical |
| Preprocessing of predictors | Description of any preprocessing of distribution shape of predictors samples, such as normalization, transformations, over- or under-sampling or adjusting between cohort samples. | Description |
| Sample Size | | |
| Sample Size calculation and statistical power | Y/N. Prior study the minimum required size was calculated to assess enough statistical power. | Logical |
| Number of participants | Nº participants participating in study/trial. | Numerical |
| Nº samples with outcome | Nº samples with the outcome (positive/target class in classification) | Numerical |
| Missing data | | |
| Is missing data mentioned? | Y/N. Whether some missing data, samples or variables were mentioned or not. | Logical |
| Nº Samples with missing data | N/A if not described. | Numerical |
| Handling of missing data | Method or algorithm applied to fix possible bias due to missingness. | Description |
| Handling method | Method category to eliminate bias from missing data. Exclusion, single imputation, multiple imputation, manual revisión… | Categorical |
| Model development | | |
| Type of modelling | Supervised, semi-supervised, unsupervised, reinforce, … | Categorical |
| Type of model | Specify the algorithm family (i.e. regression, Bayesian, SVM, CNNs, …) | Description |
| Method for selection of candidate predictors | Feature selection method. | Description |
| Shrinkage of predictors | If predictors were biased or weighted by any method to increase performance (not samples, but specific predictors). | Description |
| Model Performance | | |
| Calibration | Methods used to assess if and calibrate the model (i.e. calibration plot, calibration slope, Hosmer-Lemeshow test) | Description |
| Classification/Discrimination Measures | I.e. S, Sp, AUC, C-statistic, Accuracy, R2, RMSE, … | Description |
| Model significance | Y/N. Whether model significance was tested or not (i.e. permutation tests, …) | Logical |
| Model Evaluation | | |
| Type Validation | If performance of the model was tested in the same dataset (internal validation) - it generally means a risk of introducing bias due to same statistical distribution – or using a different dataset retrieved anywhere. | Categorical |
| Method for testing model | Method category used to test the final reported performance metrics (not for tuning or evaluating different models). Hold-out, cross-validation, external evaluation, … | Description |
| Discussion | | |
| Interpretation of models | Y/N. If the performance and operation of the model was described in discussion section. (i.e. why X predictors were chosen, which implications can be drawn from the model, why model performance was either good or bad, … NOT just describing and validating the metrics) | Logical |
| Comparison with other models | Y/N. If the reported model was compared with other models (i.e. performance of models which were tried for same goal, NOT comparing consequences of the model with literature). | Logical |

AI models were evaluated according to the purpose for which they were applied by the researchers in the study. Six categories were defined to classify the studies:

- - Predictor identification: The study aims to identify best predictors for a specific outcome or condition defined in the model. This means, the goal of the study was to extract the top predictors the AI model used to reach the ground truth.
  - Subgroups / patterns identification: The study aims to identify clusters or classify data according to data associations.
  - Forecasting symptoms: The study goal is forecasting symptom severity for a specific disease in the future.
  - Treatment quality: The study evaluates the quality and specificity of a treatment for individuals or compare performance of some treatments.
  - Prognosis: The purpose of the AI in the study is predicting the risk of a specific disorder, condition or event in future, as continuous or categorical outcome.
  - Diagnose: The study aims to distinguish target patients of a disease among all participants / samples.

## S3. Evaluation of risk of bias

PROBAST tool (Prediction model Risk Of Bias ASsessment Tool) guidelines was applied as one of the best standards guidelines for analysing the risk of bias and applicability of reviewed AI models. However, due to the nature of this systematic review, the scope of the PROBAST tool was partially limited for the full contextual analysis on possible biases. Mostly, focusing on the context of the clinical trial and quality of the data collection methodology. Thus, the first domain – *Participants* – was extended based on Cochrane tool for Randomized Clinical Trials. Moreover, some categories in *Predictors*, *Outcome* and *Analysis* were not applied due to be more suitable for a deeper analysis in a review for a specific outcome condition or modelling that is beyond the scope of this review. These categories are the one listed in PROBAST publication as 2.2, 3.2, 3.3, 3.6, 4.3, and 4.6.

| Risk of bias *Participants* | |
| --- | --- |
| Were appropriate data sources used? | Yes, no or unclear. Evaluation of data collection methods and study design based on the outcome and purpose of the AI in the study. |
| Was the collection of data blinded when it should be? | Yes, no or unclear. Assessed if the blindness was ensured when it was needed to reduce the risk of bias in collection and evaluation of participants outcomes and data. |
| Is it unlikely to introduce some bias in the distribution of samples due to missing outcome data or exclusion criteria? | Yes, no or unclear. Answer no if it was possible to lose information or do not have a suitable data for real world due to trial issues such as loss of follow-up, inclusion & exclusion criteria or bad designs of cross-sectional studies. |
| Was the number of samples enough to ensure a significative performance of the models? | Yes, no or unclear. Answer ‘Yes,’ if the number of participants and samples were enough to have a model trained with significant data. Reported calculation of statistical power of the sample was evaluated positively. |
| Risk of bias *Participants* | Low, high or unclear, based on the reported previous answers. |
| Risk of bias *Candidate predictors* | |
| Were predictors defined and assessed in a similar way for all participants? | Yes, no or unclear. Evaluating the reporting of how candidate predictors were collected. |
| Are all predictors available at the time the model is intended to be used? | Yes, no or unclear. Will predictors and data be available to generalise the model in clinical practice? |
| Risk of bias *Candidate predictors* | Low, high or unclear, based on the reported previous answers. |
| Risk of bas in *Outcome definition* | |
| Was the outcome determined appropriately? | Yes, no or unclear. Was the outcome defined and was the collection method appropriate? |
| Was the outcome defined and determined in a similar way for all participants? | Yes, no or unclear. Was the same definition for the outcome similarly applied and extracted for all patients? |
| Was the outcome determined without knowledge of predictor information? | Yes, no or unclear. Was the outcome assessed using information from the candidate predictors? Is it possible to introduce bias due to unintentionally fitting the outcome and predictors? |
| Risk of bas in *Outcome definition* | Low, high or unclear, based on the reported previous answers. |
| Risk of bias in *AI analysis* | |
| Were there a reasonable number of participants with the outcome? | Yes, no or unclear. Was a reasonable number of participants and samples with the outcome? Was a significant imbalance of classes in case of classification? |
| Were continuous and categorical predictors handled appropriately? | Yes, no or unclear. Were candida predictors properly preprocessed? The preprocessing steps could change depending of the AI algorithm and its mathematical bases. |
| Were participants with missing data handled appropriately? | Yes, no or unclear. Were missingness accounted for reduce alterations or bias in data distribution? Were possible missing data imputation methods properly evaluated and applied? |
| Was selection of predictors carried on with enough stastical power? | Yes, no or unclear. Did the feature selection method account for the true relations between candidate predictors and the outcome? Was univariate analysis avoided? Was feature selection performed when needed? |
| Were relevant model performance measures evaluated appropriately? | Yes, no or unclear. Were enough performance metrics extracted and evaluated to analyse properly the model performance? Were they evaluated appropriately? |
| Were model overfitting and optimism in model performance accounted for? | Yes, no or unclear. How were the validation strategies to reduce model optimism? Was external validation applied to evaluate the real performance of the model? |
| Were final predictors and weights reported? | Yes, no or unclear. Were at least top predictors reported? Were the predictor importance altered by shrinkage or weights? |
| Were model features and coefficients reported? | Yes, no or unclear. Were hyperparameters of the model reported? Were trained model coefficient reported? |
| Risk of bias in *AI analysis* | Low, high or unclear, based on the reported previous answers. |

## S4. Data collection for AI applications

| **Category** | **Number of AI studies** | **Proportion** |
| --- | --- | --- |
| **Biomarkers/predictors** | **28** | **18.30%** |
| **Retrospective collection** | **24** | **15.69%** |
| Case-control | 1 | 0.65% |
| Longitudinal naturalistic study | 2 | 1.31% |
| non-randomized Clinical Trial | 1 | 0.65% |
| Not reported / Unclear | 2 | 1.31% |
| Prospective cohort | 5 | 3.27% |
| RCT | 13 | 8.50% |
| **Prospective collection** | **4** | **2.61%** |
| Case-control | 1 | 0.65% |
| Cross-sectional | 2 | 1.31% |
| Prospective cohort | 1 | 0.65% |
| **Diagnose** | **20** | **13.07%** |
| **Retrospective collection** | **12** | **7.84%** |
| Case-control | 4 | 2.61% |
| Not reported / Unclear | 1 | 0.65% |
| Prospective cohort | 6 | 3.92% |
| Prospective cohort + case-control | 1 | 0.65% |
| **Prospective collection** | **8** | **5.23%** |
| Case-control | 1 | 0.65% |
| Cross-sectional | 3 | 1.96% |
| non-randomized Clinical Trial | 2 | 1.31% |
| Prospective cohort | 1 | 0.65% |
| RCT | 1 | 0.65% |
| **Forecasting symptons** | **7** | **4.58%** |
| **Retrospective collection** | **4** | **2.61%** |
| Not reported / Unclear | 1 | 0.65% |
| RCT | 3 | 1.96% |
| **Prospective collection** | **3** | **1.96%** |
| Cross-sectional | 1 | 0.65% |
| Prospective cohort | 1 | 0.65% |
| RCT | 1 | 0.65% |
| **Prognosis** | **23** | **15.03%** |
| **Retrospective collection** | **18** | **11.76%** |
| Longitudinal naturalistic study | 7 | 4.58% |
| Prospective cohort | 9 | 5.88% |
| RCT | 2 | 1.31% |
| **Prospective collection** | **5** | **3.27%** |
| Case report | 1 | 0.65% |
| Case-control | 1 | 0.65% |
| Prospective cohort | 3 | 1.96% |
| **Subgroups/patterns identification** | **31** | **20.26%** |
| **Retrospective collection** | **20** | **13.07%** |
| Case-control | 1 | 0.65% |
| Cross-sectional | 3 | 1.96% |
| Longitudinal naturalistic study | 5 | 3.27% |
| Prospective cohort | 3 | 1.96% |
| RCT | 7 | 4.58% |
| RCT + Cohort | 1 | 0.65% |
| **Prospective collection** | **11** | **7.19%** |
| Cross-sectional | 5 | 3.27% |
| Longitudinal naturalistic study | 1 | 0.65% |
| non-randomized Clinical Trial | 2 | 1.31% |
| RCT | 3 | 1.96% |
| **Treatment quality** | **44** | **28.76%** |
| **Retrospective collection** | **32** | **20.92%** |
| Case report | 1 | 0.65% |
| non-randomized Clinical Trial | 2 | 1.31% |
| Prospective cohort | 1 | 0.65% |
| RCT | 28 | 18.30% |
| **Prospective collection** | **12** | **7.84%** |
| non-randomized Clinical Trial | 8 | 5.23% |
| RCT | 4 | 2.61% |

## S5. Risk of bias stratified by disorders, study designs and outcome

The risk of introducing bias was evaluated for each AI study in 4 categories: data collection, candida predictors, outcome definition and AI analysis. Here, some insights about the analysis in risk of bias are given with more details. Risk of bias for AI studies related to depression or related disorders (N=70), Schizophrenia and primary psychotic disorders (N=26) and Disorders specifically associated with stress (N=12) were evaluated as the major categories to assess if this risk vary depending of the type of disorder research (figure Appendix V A). Also studies were grouped in the major categories for study design as well as for purpose of the AI application (figure Appendix V B).


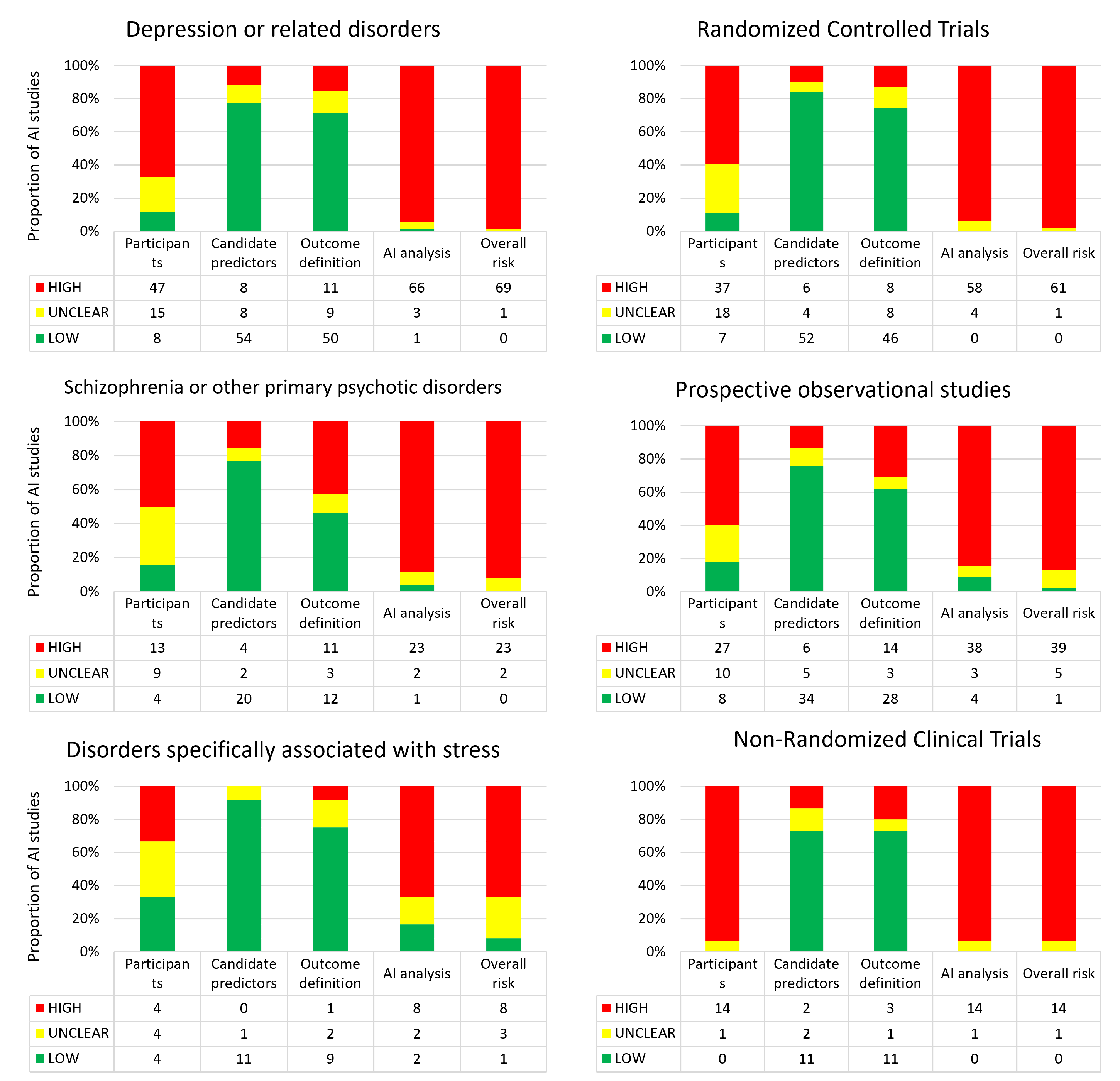


Figure Appendix V A. Risk of bias stratified by ICD-11 categories disorders (left charts) and main types of study design (right charts). Depressive or related disorders (N=70), Schizophrenia and primary psychotic disorders (N=26) and Disorders specifically associated with stress (N=12). RCTs (N=62), Prospective observational studies – i.e. Prospective cohorts and longitudinal studies – (N=45) and non-randomized (interventional) clinical trials (N=15).


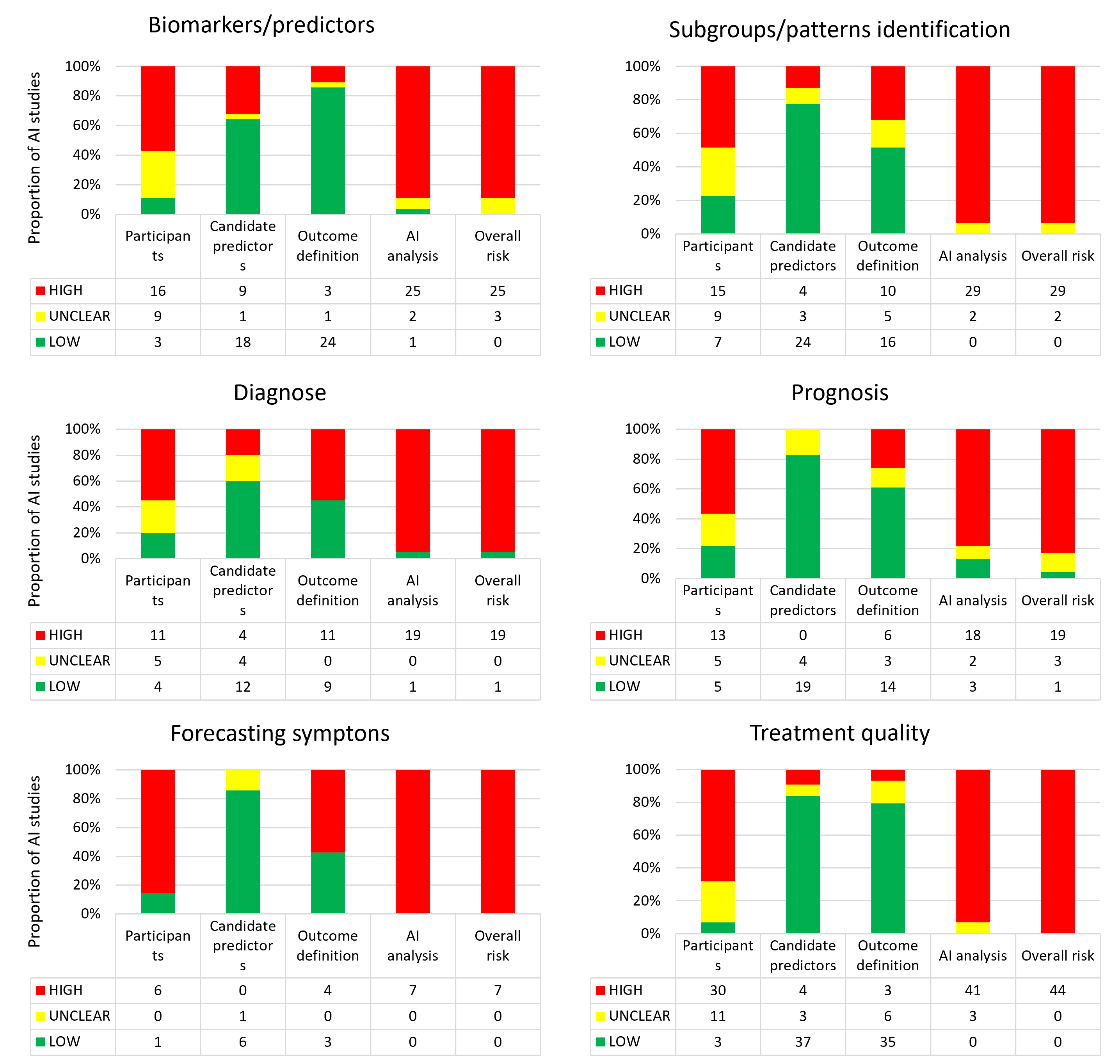


Figure Appendix V B. Risk of bias stratified by Purpose of AI models. Treatment quality (N=44), followed by Subgroups/patterns identification (N=31), Predictor identification (N=28), Prognosis (N=23), Diagnose (N=20), and Forecasting symptoms (N=7).
